# A hashtag recommendation system for twitter data streams

**DOI:** 10.1186/s40649-016-0028-9

**Published:** 2016-05-31

**Authors:** Eriko Otsuka, Scott A. Wallace, David Chiu

**Affiliations:** 1 School of Engineering and Computer Science, Washington State University, Vancouver, USA; 2 Department of Mathematics and Computer Science, University of Puget Sound, Tacoma, USA

**Keywords:** Twitter, Recommendation

## Abstract

**Background:**

Twitter has evolved into a powerful communication and information sharing tool used by millions of people around the world to post what is happening now. A hashtag, a keyword prefixed with a hash symbol (#), is a feature in Twitter to organize tweets and facilitate effective search among a massive volume of data. In this paper, we propose an automatic hashtag recommendation system that helps users find new hashtags related to their interests on-demand.

**Methods:**

For hashtag ranking, we propose the Hashtag Frequency-Inverse Hashtag Ubiquity (HF-IHU) ranking scheme, which is a variation of the well-known TF-IDF, that considers hashtag relevancy, as well as data sparseness which is one of the key challenges in analyzing microblog data. Our system is built on top of Hadoop, a leading platform for distributed computing, to provide scalable performance using Map-Reduce. Experiments on a large Twitter data set demonstrate that our method successfully yields relevant hashtags for user’s interest and that recommendations are more stable and reliable than ranking tags based on tweet content similarity.

**Results and conclusions:**

Our results show that HF-IHU can achieve over 30 % hashtag recall when asked to identify the top 10 relevant hashtags for a particular tweet. Furthermore, our method out-performs kNN, k-popularity, and Naïve Bayes by 69, 54, and 17 %, respectively, on recall of the top 200 hashtags.

## Background

Micro-blogging has become a popular communication and information search tool, and Twitter is one of the most prevalent micro-blogging platforms today, with over 200 million active users posting *tweets*, a message limited to 140 characters [1]. The tweet’s character limit promotes users to casually update posts, whereas traditional blogging has a tendency to require more dedicated time to write a new post. Additionally, with increasing ownership of mobile devices, many users are engaged to Twitter activities, resulting in over 400 million tweets sent to the Twitter network per day [2, 3]. The downside to this popularity is that Twitter users may easily be overwhelmed by the massive volume of data. As a mechanism to combat the issue, Twitter users have organically incorporated the hashtag culture into their tweets. A *hashtag* is a word or a phrase without spaces prefixed with the hash symbol *#* inserted anywhere in the body of tweets. Trendy topics can be quickly propagated among millions of users through tagging, which creates an instant community with similar interests. With the implementation of the hashtag search feature in Twitter, many individual users and business marketers have started applying tagging to organize posts into related conversations and facilitate easier search by associated hashtags.

As tagging culture becomes widely adopted, the development of hashtag recommendation systems has gained researchers’ attention. Some recent studies have proposed to recommend predefined hashtags [4, 5] or general topics hidden in each tweet [6]. Though these systems are beneficial in encouraging and assisting users to get into the tagging habit, it may not be sufficient for information seekers who wish to find newly emerging hashtags. In contrast, recommending the most popular hashtags does reflect timely topics, but it often includes heavily used general hashtags and suggestions are not personalized. Other studies have proposed recommending hashtags based on similar tweets [7].

In this paper, we propose a new method to automatically recommend personalized trending hashtags based on users’ tweets. Our approach does not limit the number of candidate hashtags to be examined, but rather provides an arbitrarily long list of ranked recommendations. Specifically, we make the following contributions:

- We build an effective hashtag recommendation system using a proposed hashtag ranking method, *Hashtag Frequency-Inverse Hashtag Ubiquity* (HF-IHU).
- We provide scalable Map-Reduce algorithms to construct two fundamental structures, the term-frequency map for hashtags (THFM) and hashtag-frequency map (HFM). These indices are used to support fast HF-IHU calculations.
- We conduct a nuanced evaluation of HF-IHU over a large Twitter data set. We compare HF-IHU against several popular schemes, including *k*-nearest neighbors using Cosine similarity, *k*-popularity, and Naïve Bayes. Our results show that HF-IHU achieves substantially higher recall than the other schemes and is resistant to retweets.

The remainder of this paper is organized as follows. “Background” section provides an overview of Twitter terminology. “Index generation and ranking” section describes our distributed Map-Reduce algorithm for building the inverted indices that are central to our recommendation algorithm. Our hashtag ranking algorithm is also presented in this section. In “Experimental evaluation” section, we describe our experimental setup and the performance results of our algorithm. Finally, in “Conclusion and future work” section, we conclude the paper with a discussion and ideas for future work.

## Background

This section introduces the terminologies that are used to address the services and features of Twitter.

- Tweet: In Twitter, a *tweet* is a short message limited to 140 characters posted by a user. *To tweet* is also used as a verb for posting such messages. Unless an account is private, all tweets are public by default.
- Follow: In Twitter, subscribing to other users to read their tweets is called a *follow*. Users can become a *follower* of someone without his or her approval unless the user has set tweet protection or have blocked the user. Unlike *friend-ing* in other social networks, following is not mutual.
- User: A user is identified by a Twitter handle, *@user*. Users can *mention* other users by adding another their handle in their tweet. When mentioned, a user is notified by the Twitter API, and the mentioning tweet is displayed on the user’s feed.
- Retweet: When users want to share someone else’s tweet, they can *retweet* it to their own followers. There are two ways: *automatic retweeting* and *manual retweeting*. Automatic retweeting is Twitter’s built-in feature where a tweet is shared verbatim and marked as a retweet. Users can manually retweet by copying the body of a tweet they want to share and pasting it into their tweet box. Since these tweets are not automatically marked as retweets, Twitter instructs users to add the keyword *RT* and the initial author’s handle in the tweet content. Sometimes, users will add their own thoughts to a manual retweet, which changes the content of the original tweet.
- Hashtag: A hashtag is a keyword prefixed with *#* and can be placed anywhere in the body of the tweet to categorize or mark words/phrases as keywords related to their tweets. By clicking hashtags in Tweets, users can view all Tweets containing the hashtag. Extremely popular hashtags often become trends.
- Trend: Twitter displays a list of immediately popular keywords and hashtags on the user’s homepage to help users discover the emerging topics in Twitter, and these keywords are referred to as *trends*. Trends are user-locality aware, but are not context-sensitive.

## Index generation and ranking

Our hashtag ranking algorithm is inspired by the well-known TF-IDF [8] approach used in information retrieval. Our algorithm relies on two central data structures that are compiled from a large number of Tweets. We built these data structures using the Hadoop distributed computing platform with Map-Reduce [9].

**Fig. 1:**
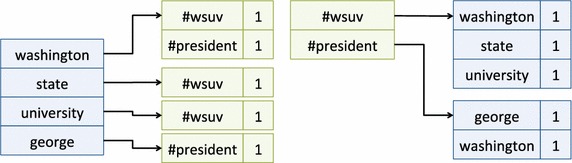
THFM (*left*) and HFM (*right*)

Both data structures are nested maps: the first is a term to hashtag-frequency-map (THFM); the second is the converse—a hashtag frequency-map (HFM). In the THFM, the primary keys are terms that have been observed in tweets. The value associated with each primary key is a map from hashtag to a frequency count indicating how often that hashtag (the secondary key) has occurred with the term specified by the primary key. The HFM is an analogous data structure using hashtags as the primary key and term frequencies as the final value. Figure 1 illustrates the THFM (left) and HFM (right) provided a data set containing two tweets: “washington state university #wsuv” and “george washington #president”.

Given that 400 million tweets are posted per day [3], the THFM and HFM need to be updated frequently so that the recommendation of hashtags reflects the most recent trends. Moreover, simply processing the tweets over a relatively small window of time (e.g., weeks) requires substantial computational power. As a result, we have developed an approach for building the THFM and HFM using the Map-Reduce programming paradigm [9] that can scale easily to hundreds of nodes.

### Map-Reduce

The Map-Reduce model consists of three phases: *Map*, *Shuffle* and *Reduce*. Map and Reduce are user-provided functions, but Shuffle is performed automatically by the Map-Reduce framework between the Map phase and the Reduce phase. Given a data set, the master node divides the problem into data-parallel sub-problems and distributes them to worker nodes. At each worker node, the Map function produces a set of key-value pairs as intermediate outputs. Shuffle collects the intermediate outputs from the Map function and groups them by key. Worker nodes are then assigned with the grouped outputs and perform the Reduce task to process the final results.

**Fig. 2:**
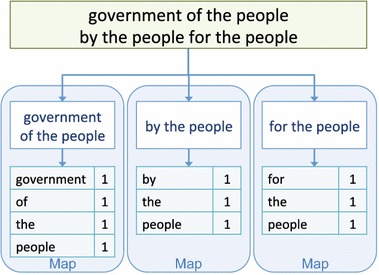
Map-Reduce—Map phase

**Fig. 3:**
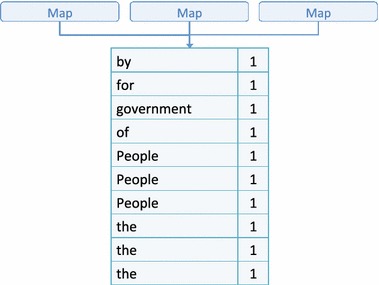
Map-Reduce—Shuffle phase

Provided the input “*government of the people by the people for the people*”, Fig. 2 illustrates a simple example of Map-Reduce that computes the term-frequency of the input. In the Map phase, instead of performing the Map function on the entire input, the input is divided into sub-problems and distributed to worker nodes. The Map function for this example outputs key-value pairs using terms as keys and ‘1’ as values. The outputs from Map are fed into the Shuffle phase as shown in Fig. 3. Shuffle sorts the data by key (terms in this example) and sends the sorted data to the Reduce phase.

**Fig. 4:**
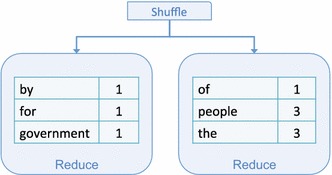
Map-Reduce—Reduce phase

In the Reduce phase, each reduce task runs the user-provided function. The received input results are combined by key and the final output includes one value per key. In this example, the Reduce function sums the value, ‘1’, for each key to generate a term frequency for the sample input. Figure 4 depicts the output from the Reduce phase for the example.

### Frequency map generation algorithm

The Map functions used to generate the THFM and HFM are similar to the Map function in the above Map-Reduce example. Provided a training data set containing a list of tweets, the program prints lines in the format of #hashtag:term, where #hashtag is a hashtag appeared in a tweet and term is a term appeared in a tweet with the hashtag. For example, when the input tweet contains multiple hashtags such as washington state university #wsuv #cs, then the result prints six lines: #wsuv:washington, #wsuv:state, #wsuv:university, #cs:washington, #cs:state, #cs:university. The Map function to generate the HFM is shown in Algorithm 1.

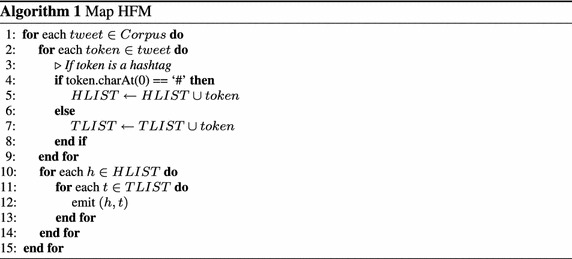

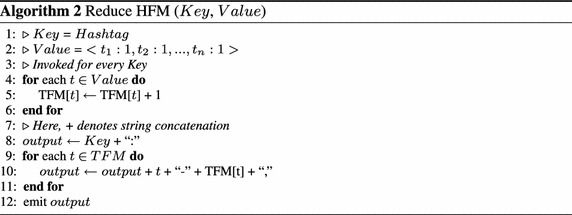





The output of the Map function is sent to the Shuffle phase and gets sorted by hashtag. Next, the reduce function (Algorithm 2) receives the shuffled key-value pairs and iterates values (terms) per key (hashtag) summing the occurrences of terms for each key. The output of the reduce function lists keys followed by key-value pairs (term to term-frequencies). The THFM is generated in an analogous manner.

The generated THFM and HFM are stored in an inverted index. Provided a term *t*, *THFM[t]* retrieves a collection of hashtags that appeared with *t* in the corpus in *O*(1) time. The frequency of each hashtag co-occurring with *t* is retrieved by *THFM[t][h]*.

Similarly, provided a hashtag *h*, *HFM[h]* contains a collection of terms that appeared with *h* in the corpus, and *HFM[h][t]* retrieves the frequency of each term co-occurring with *h*.

### Ranking hashtags with HF-IHU

After generating THFM and HFM, the next step is to score hashtags in the data set to find personalized recommendations for a user. Our proposed scoring method utilizes the variation of the TF-IDF scheme, we call Hashtag Frequency-Inverse Hashtag Ubiquity (HF-IHU). HF-IHU has two opposing weighting factors: the first is the frequency with which a hashtag appears with a given term (the hashtag frequency). The second is the hashtag ubiquity which discounts hashtags that are prevalent in all contexts and rewards hashtags that are tightly associated with a narrow subset of terms.

Provided a term *t* and a hashtag *h* which co-occurred with *t*, $hf_{t,h}$ is expressed as follows:

1 $$\begin{aligned} hf_{t,h} = \dfrac{\text{THFM}[t][h]}{\displaystyle \sum \limits _{h'} \text{THFM}[t][h']}, \end{aligned}$$

where THFM[*t*][*h*] denotes the occurrences of *h* with *t* in the corpus. The denominator of $hf_{t,h}$ is the sum of all hashtag frequencies associated with *t*. Thus, $hf_{t,h}$ measures the association between a term and a hashtag. Intuitively, if many users used a hashtag with a particular term, the hashtag is more likely relevant to the term.

The $ihu_{h}$ is derived from the following formula:

2 $$\begin{aligned} ihu_{h} = \log {\dfrac{|\text{Corpus}_{NH}|}{\displaystyle \sum \limits _{t'} \text{HFM}[h]}}, \end{aligned}$$

where $|\text{Corpus}_{NH}|$ denotes the number of all terms in the corpus with hashtags removed. The denominator of $ihu_{h}$ is the sum of all term frequencies associated with *h*. Thus $ihu_{h}$ decreases as the hashtag *h* becomes associated with a large fraction of terms in the corpus. The intuition is that these ubiquitous tags are less likely to be personally important to any given user, thus they must overcome a larger hurdle than other hashtags to be recommended. This is in contrast to the IDF term in the well-known TF-IDF, where IDF would have decreased the important of term *t*, rather than *h*, contradicting our objective.

Our main hashtag scoring algorithm is shown in Algorithm 3. The algorithm inputs a tweet, which is a list of terms $T = (t_1, ..., t_n)$. For each term $t_i \in T$, we locate all hashtags $h_j$ that co-occurred with it from our THFM and HFM indices. The hashtag-term frequency $hf_{t_i,h_j}$ and the inverse hashtag ubiquity metric $ihu_{h_j}$ are computed across all hashtags to calculate the partial score. These partial scores are aggregated for all hashtags pertaining to $t_i$ before being returned.

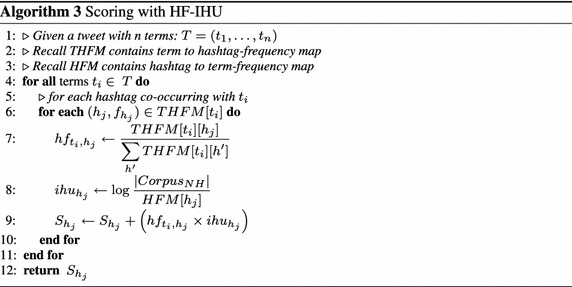



One advantage of HF-IFU is that our algorithm looks up candidate hashtags using the terms in a candidate tweet. This means that the number of candidate hashtags grows as a function of the number of terms in the tweet (t) and is bound from above by the total number of hashtags in the corpus (H). Because Twitter limits tweets to 140 characters, only a small number of words will actually fit in a tweet. Thus, the dominating term will be, in the worst case, the search over all H hashtags. However, the terms in any given tweet will typically not be associated with all H hashtags, so in practice, we would expect the cost to be substantially lower.

It is also worth noting, that HF-IFU avoids a search over all terms in the corpus. The best approaches for *k*-Nearest Neighbors, another plausible method for generating recommendations, are linear in the dimensionality of the data (e.g.,  [10–12]). In our domain, tweets are represented with a bag-of-words model which is a vector whose dimensionality is exactly the size of the entire dictionary of known terms. This is a problem for kNN search, since we expect that the total number of terms will be substantially larger than H (the number of hashtags). Worse, there is no short-cut in a search over *d*-dimensions as there is in our approach when a term is associated with only a few hashtags. Rather, for kNN search, all searches will exhibit the worst-case linear in *d* cost. Thus, a search that examines all H hashtags is much preferable, from a cost perspective, to one that must examine all *d* dimensions of the data set.

## Experimental evaluation

In this section, we present a nuanced evaluation of our system. We initially describe the characteristics of the Twitter corpus we obtained and will use for evaluation in "Tweet corpus" section. Next, in "Experimental setup" section, we explain how we will carry out the results, as well as the algorithms we compare against. The recall metric to judge the goodness of the hashtag ranking algorithms is described in "Evaluation metric: precision and recall" section. Finally, detailed results of our evaluation, including a case study, are then presented in "Experimental results" section.

### Tweet corpus

To evaluate our hashtag recommendation system, we first obtained the Tweets 2011 corpus, consisting of a collection of tweet identifiers, provided by Twitter for the TREC 2011 Microblog Track 2011 [13]. The corpus represents approximately 13 million tweets sampled between January 23 and February 5, 2011. We used the HTML crawler from an open-source tool^1^ and directly downloaded the actual tweets from Twitter including id, username, timestamp, HTTP response code, and tweet body.

Although the tool was supplied with the repair code to re-fetch missed tweets, the crawler still returned some null tweets after several attempts to re-fetch them. Most of the missed tweets are returned with HTTP status code 301, 302 or 404. The description of code 301 is moved permanently and that of code 302 is moved temporarily, and both denote retweets, which is not completely handled by the tool. Code 404 means that the requested page was not found, which denotes deleted tweets. In all, the corpus size was 3 GB.

**Fig. 5:**
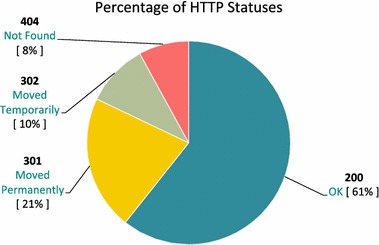
HTTP Response Code

**Fig. 6:**
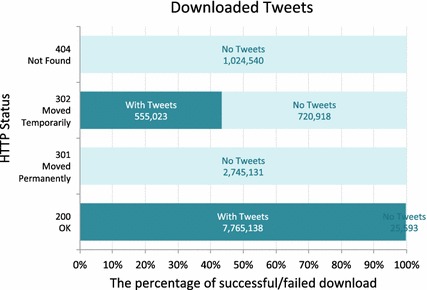
Downloaded Tweets

As illustrated in the figures below, approximately $61\,\%$ of the provided corpus included tweet bodies, and 8.3 million tweets were successfully retrieved to be used in the experiment. Figure 5 depicts the distribution of tweets with returned HTTP statuses in the downloaded data, and Fig. 6 shows the number of retrieved tweet bodies per HTTP status code.

Twitter users often *mention* one or more users in their own tweets with *@user* to include the other users in their conversation. Although mentions appear many times in the data set, we removed these user handles from the data set because they are generally used to show interest in the mentioned user or the relationship, but not in the user itself. Additionally, we follow common information retrieval preprocessing steps by: (1) removing punctuation and non-alphanumeric symbols; (2) removing common stop-words; (3) transforming all text to lowercase; (4) stemming (we employed the Porter Stemmer from the open source Python library NLTK [14]). Some non-English languages that do not use word dividers (e.g., a blank space between words) such as Japanese would require an extra pre-processing step to identify the parts of speech before performing the above steps.

We successfully downloaded approximately 8.3 million tweets. Text pre-processing eliminated 1 % of these tweets because some consisted of only stop words or user mentions. We then found that remaining tweets, approximately 13 %, contained at least one hashtag. The remaining 87 % contained no hashtags. An overview of the downloaded data characterization is listed in Table 1.

**Table 1:** Overview of data characterization

Characteristic	Value
Downloaded tweets	8,320,161
Cleaned tweets	8,234,098
Tweets containing no hashtags	7,182,506
Tweets containing at least one hashtag	1,051,592
One hashtag per tweet	827,630
Two hashtags per tweet	145,718
More than two hashtags per tweet	78,244
Maximum number of hashtags used in a tweet	28

### Experimental setup

To evaluate the performance of our recommendation method, we set up a data set by splitting the pre-processed data into a training set (90 %) and a test set (10 %). The training set contains approximately 8.1 million tweets; roughly 900,000 of these contain at least one hashtag. The test set contains about 100,000 tweets, all of which include at least one hashtag. Finally, the THFM and HFM were generated by running our Map-Reduce applications using the training set as input.

To evaluate HF-IHU, we compared it with four other recommendation methods: Cosine similarity with *k*-Nearest Neighbour (kNN), overall popularity, and Naïve Bayes, and User similarity and Tweet similarity. The descriptions of each tested method are briefly explained below:

- *kNN with Cosine similarity* Provided a tweet in the training set, $t_1$ and another tweet $t_2$ from the test set, this method computes the Cosine similarity:
  3 $$\begin{aligned} \cos (t_1,t_2) = \frac{t_1 \cdot t_2}{\parallel t_1 \parallel \parallel t_2 \parallel }. \end{aligned}$$
  For each tweet in the test data, we iterated through all tweets in the training data and computed the Cosine similarity between them. We found the *k*-Nearest Neighbors $(k=200)$ of the test tweet and used these neighbors to produce a ranked list of recommended hashtags.
- *Naïve Bayes* This method makes recommendations based on the results of a multinomial Naive Bayes model that is standard for text documents with large vocabularies and sparse data. In this model, the hashtag ranking depends on the posterior probability of a hashtag $H_i$ given a tweet composed of a set of terms $t_j$ each with frequency $f_{t_j}$:
  4 $$\begin{aligned} P(H_i|t_1, ...,t_n) \propto P(H_i)\prod _jP(t_j|H_i)^{f_{t_j}}. \end{aligned}$$
  We use Laplacian smoothing to deal with edge conditions in the conditional probability tables.
- *Overall popularity* This method simply recommends the most frequently occurring (popular) hashtags in the training set for each test tweet. Table 2 shows the top 30 popular hashtags in our data set. This ranking method is not designed to make personalized recommendation, and therefore, the recommendations are consistently the same hashtags for any given tweet.
- *User similarity and Tweet similarity* This method is proposed by Kywe et al. [15]. They score candidate hashtags based on the combination of user similarity and tweet similarity employing TF-IDF as a means of scoring each similarity. A user is represented by the *preference weight* for each hashtag in the data. The preference weight $w_{ij}$ for user $u_j$ for hashtag $h_i$ is defined by the following formula:
  5 $$\begin{aligned} w_{ij}&= \text{TF}_{ij} \cdot \text{IDF}_i ,\end{aligned}$$
  6 $$\begin{aligned}&= \frac{\text{Freq}_{ij}}{\text{Max}_j} \cdot \text{log} \left( \frac{N_u}{n_i}\right), \end{aligned}$$
  where $\text{Freq}_{ij}$ is the usage frequency of hashtag $h_i$ by $u_j$, $\text{Max}_j$ is the total number of hashtags used by $u_j$, $N_u$ is the total number of users, and $n_i$ denotes the number of users who used $h_i$. Similar tweets are retrieved in a similar manner as shown in the following formula:
  7 $$\begin{aligned} w_{kl}&= \text{TF}_{kl} \cdot \text{IDF}_l\end{aligned}$$
  8 $$\begin{aligned}&= \frac{\text{Freq}_{kl}}{\text{Max}_k} \cdot \text{log}\left( \frac{N_t}{n_l}\right), \end{aligned}$$
  where $\text{Freq}_{kl}$ is the frequency of word $w_l$ in tweet $t_k$, $\text{Max}_k$ is the total number of word used in $t_k$, $N_t$ is the total number of tweets, and $n_l$ denotes the number of tweets in which $w_l$ appears. To find the top *X* similar users, *HTofUsers*(*u*), the cosine similarity between a target user *u* and another user $u_i$, is measured as follows:
  9 $$\begin{aligned} \cos (u,u_i) = \frac{u \cdot u_i}{\parallel u \parallel \parallel u_i \parallel }. \end{aligned}$$
  Similarly, to find the top *Y* similar tweets, *HTofTweets*(*t*), the cosine similarity between a target tweet *t* and another tweet $t_k$, is measured as follows.
  10 $$\begin{aligned} \cos (t,t_k) = \frac{t \cdot t_k}{\parallel t \parallel \parallel t_k \parallel }. \end{aligned}$$
  After finding *HTofUsers*(*u*) and *HTofTweets*(*t*), the candidate hashtags for the target tweet *t* posted by user *u* are obtained in the following formula:
  11 $$\begin{aligned} SuggestedHashtags(u,t) = HTofUsers(u) \cup HTofTweets(t). \end{aligned}$$
  The recommendations are ranked by hashtag frequency in *SuggestedHashtags*(*u*, *t*). Since they reported that this method performed the best when $X=5$ and $Y=50$, we used the same numbers for these parameters in our experiment.

**Table 2:** Top 30 popular #hashtags

No.	Hashtag	No.	Hashtag	No.	Hashtag
1	#ff	11	#bbb	21	#nicovideo
2	#egypt	12	#news	22	#1
3	#jan25	13	#icantdateyou	23	#partiu
4	#nowplaying	14	#fail	24	#shoutout
5	#np	15	#sougofollow	25	#music
6	#mentionke	16	#sotu	26	#followme
7	#fb	17	#rt	27	#follow
8	#jobs	18	#tcot	28	#win
9	#teamfollowback	19	#famouslies	29	#nw
10	#followmejp	20	#improudtosay	30	#iphone

### Evaluation metric: precision and recall

To evaluate the performance of the above methods, we examined each method’s ability to recall hashtags from our ground-truth tweets in the test set. We view recall as the most salient metric since our approach is designed to recommend new hashtags, which by definition may not be present in the original tweet. For completeness, however, we also include the precision for each method. High precision is obtained if the recommended hashtags are exactly those found in the original tweet; novel recommendations decrease precision but not recall.

Formally, let $T = \{T_1, \ldots, T_n\}$ denote the set of *all* tweets in our test set. Each tweet $T_i$ is composed of a set of terms, $T_i = \{t_1, \ldots, t_m\}$ and a set of hashtags $H_i = \{h_1, \ldots, h_k\}$. For each method, we input a tweet $T_i$ to produce a set of ranked *recommended* hashtags $S_i = \{s_1, \ldots, s_p\}$ for that tweet. We then compare the recommended hashtags $S_i$ to the ground-truth hashtags, $H_i$, that we removed from the original tweet. A well-functioning recommendation system will generate $S_i$ such that most, or all, of the hashtags from $H_i$ are highly ranked. To measure this, we define:

12 $$\begin{aligned} 1_{H_i}(s_j) = {\left\{ \begin{array}{ll} 1, \quad &{} \text {if} \, s_j \in H_i\\ 0, &{} \text {otherwise}\\ \end{array}\right. } \end{aligned}$$

**Fig. 7:**
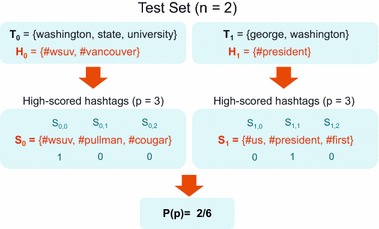
Example measurement of precision

The (micro-averaged) precision, *P*, can be computed as follows,

13 $$\begin{aligned} P(p) = \frac{\displaystyle \sum \nolimits _{i=0}^n\displaystyle \sum \nolimits _{j=0}^p{1_{H_i}(s_j)}}{\displaystyle \sum \nolimits _{i=0}^n{|S_i|}}, \end{aligned}$$

where *n* is the total number of tweets in the test set and *p* is the number of ranked recommendations provided by the method under examination. Figure 7 shows a simple example of the precision notations with a sample test set containing two tweets.

The micro-averaged recall, *R*, can be computed as follows,

14 $$\begin{aligned} R(p) = \frac{\displaystyle \sum \nolimits _{i=0}^n\displaystyle \sum \nolimits _{j=0}^p{1_{H_i}(s_j)}}{\displaystyle \sum \nolimits _{i=0}^n{|H_i|}}. \end{aligned}$$

**Fig. 8:**
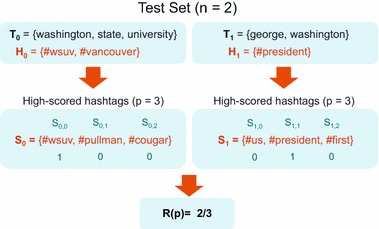
Example measurement of recall

Figure 8 shows an example of the recall notations with a sample test set of two tweets.

### Experimental results

#### Full corpus

**Fig. 9:**
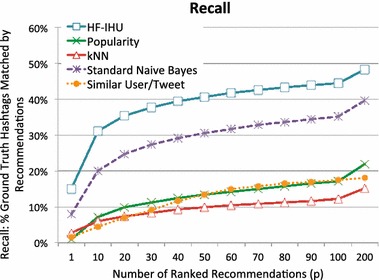
Recall for the ranking methods

**Fig. 10:**
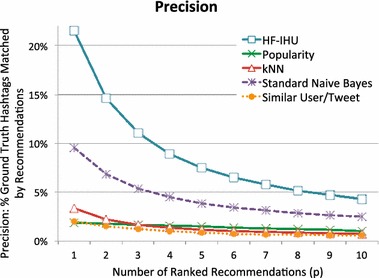
Precision for the ranking methods

We ran all the ranking methods introduced above on the full set of testing data and plotted *R* (recall) and *P* (precision) for various values of *p* (on the horizontal axis). Full recall results are shown in Fig. 9 and full precision results are shown in Fig. 10. At $p=1$, each method returns only its top recommended hashtag for each tweet in the test set, while at $p=100$ each method returns its top 100 recommended hashtags for that tweet. Note an ideal method will not be able to obtain 100 % Recall. Rather the maximum recall ceiling lies at roughly 74 % when $p=1$ and increases to approximately 81 % when $p \ge 6$. The maximum-recall ceiling lies below 100 % because not all tags in the test set occur in the training data. Thus, some tags in the test tweets could never be recommended. Moreover, since many test tweets have more than one hashtag, for small values of *p*, some tags will necessarily go unmatched, even if they would be recalled for larger values of *p*. The maximum-recall ceiling reaches an asymptote near $p=6$ since very few tweets in the test set have more than six hashtags to recall.

Figures 9 and 10 show that HF-IHU consistently reproduced the removed-hashtags over the other methods. The result of overall popularity simply reflects the percentage of popular hashtags occurring in our test set as expected. One surprise in the results is how poorly kNN performs. One of the strengths of the HF-IHU method over kNN is that it examines the weight of all of the candidate hashtags, whereas kNN only examines at the term-level and simply returns hashtags that occur with similar tweets. Thus, all hashtags in those similar tweets are ranked equally. For example, to find hashtags for a test tweet “george washington” with the HF-IHU method, it first computes the score for all hashtags that occurred with the term “george” (576 hashtags) and then computes for hashtags that occurred with “washington” (641 hashtags), accumulates hashtag scores, and it finally returns the top *n* high-scored hashtags.

With the kNN method, however, only similar tweets are used to determine recommended tags. Thus, the individual terms have little direct contribution. Rather, it is the set of terms that will determine the recommendations. For example, in our test tweet “george washington”, tweets that have exactly these terms and one or more hashtags will have a perfect similarity score while tweets that differ only in one word will be close neighbors. On the other hand, tweets that not only have both “george” and “washington”, but also contain a variety of other terms will *not* be close neighbors. The result is that fewer tweets will be taken into account to determine recommendations. This will tend to bias the statistical relationships in an unpredictable, and based on the observed results, often undesirable manner.

In Fig. 10, we show the precision results of all methods over *p*. The higher *P*(*p*) values for small values of *p* indicate that our method is selecting the hashtags from the ground-truth set, $H_i$, to be highly ranked in the result set. As expected, as *p* increases, the precision decreases across all methods. Even as the methods reach their asymptotic limits, HF-IHU consistently outperforms the other methods.

There is a need to explain the low precision results. Defined in Eq. 13, the precision *P*(*p*) is normalized over the number of hashtags returned by our system. As we ask for an increasing number of recommended hashtags (along the horizontal axis), the denominator also increases. However, recall from Table 1 that most tweets in our sanitized corpus only contain few hashtags (~1.34 hashtags per tweet on average). Worse, only $7.4 \, \%$ of tweets contain more than two hashtags. Therefore, when we request $p \, >\, p'$ hashtags to be recommended, where $p'$ is the actual number of hashtags in the tweet, the precision is artificially reduced. This is the case for all methods evaluated.

Even for low values *p* the precision is still evidently mediocre. For instance, precision tops out at ${\sim}25$ and ${\sim}15\,\%$ respectively for $p=1$ and $p=2$. Several issues contribute to this problem. First, tweets are short. Twitter imposes a maximum character limit per tweet of 140. An average tweet in our sanitized corpus has somewhere in the range of 3–10 real terms, not including any hashtags. Second, some tweets *only* contain hashtags, and lack any supporting terms. Third, because of the terms’ sparseness, there exist supporting terms that appear in the test set, but not in the training set. In combination, these factors conspire to add noise to the result set.

It should be emphasized, however, that the key objective of our system is to discover and recommend new hashtags to the user, which intuitively do not appear in the set of “ground truths” being returned.

#### Stratified retweets

As mentioned previously, Twitter users often *retweet* to share what they have read with their followers. Due to this retweet feature, our training set contains some tweets that are very similar to, or even exactly the same as, tweets in the test set. We conducted another experiment to explore how the existence of similar tweets affects the ranking performance. Recall that Twitter offers two ways of retweeting: automatic retweeting and manual retweeting. With automatic retweeting, users do not add any comments to the tweet text. Therefore, this kind of retweet adds duplicated tweets to the data set. With manual retweeting, users are allowed to add their own comments with a keyword *RT*; therefore, manually added retweets may be very similar tweets or could be duplicates once the retweeting keyword, *RT*, is removed in pre-processing phase.

To find similar tweets that are possibly retweets of test tweets, we used the result scores in the kNN method, which computed Cosine similarity between each tweet in the training set and each tweet in the test set. We then set a similarity threshold, *r*, that we use to distinguish likely retweets from non-retweets. For example, when $r=0.9$, we stratify the training set into: *retweets only (0.9)* which is the set of training tweets that have a Cosine similarity of 0.9 or greater with *some* tweet in the test set; and *no retweets (0.9)* which is the set of training tweets that have a Cosine similarity of less than 0.9 will *all* tweets in the test set. To observe the impact of Tweet similarity between the training and testing set, we examined the performance with $r=0.75$ and $r=0.9$ and compared these against the unstratified (complete) training data.

**Fig. 11:**
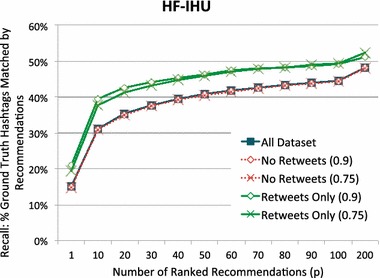
Recall depending on the number of recommended tags ranked with HF-IHU

Figure 11 shows the result of our HF-IHU ranking method on the stratified corpus. The* x*-axis in the figure represents the user-specified number of returned high-scored hashtags. The* y*-axis shows the percentage of ranked recommendations that match the removed hashtags from original tweets. Not surprisingly, HF-IHU performs better when the test set contains only retweets from the training data (Fig. 11). It is interesting to note, however, that HF-IHU’s performance on the retweet only data is typically within $7 \, \%$ of the performance on the retweet-less data and the standard unstratified corpus. This suggests that the approach is not overly sensitive to retweets and adds further evidence as to our method’s robustness.

**Fig. 12:**
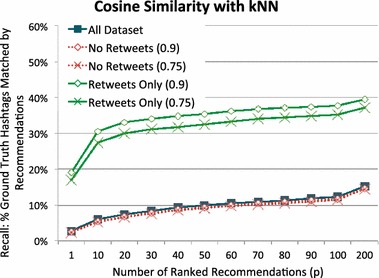
Recall depending on the number of recommended tags ranked with kNN

These findings are in contrast to the results shown in Fig. 12. Here, we see the result of running kNN on the stratified data. Previously, we noted that kNN performed notably worse than HF-IHU in terms of recall (Fig. 9). We further note that kNN is significantly influenced by the presence of retweets. The difference in performance between retweet data and retweet-less data is often more than 20 %. Interestingly, with the exception of the recall for a single recommendation (the point plotted at $x=1$), kNN’s performance on retweet only data fails to achieve the performance of HF-IHU on the full corpus.

Cosine similarity scores each training tweet only based on the similarity between each training tweet and each test tweet; hence, it simply ranks higher on retweets that presumably contain the removed hashtags. The wider gap, therefore, is not surprising. Though we expected it to show an even larger gap, the actual result is reasonable because it is not guaranteed that similar tweets are actual retweets and that similar tweets always contain all of the removed hashtags. Suppose, for example, there is a training tweet washington #wsuv and a test tweet washington #DC. Since hashtags are ignored when Cosine similarity is calculated, the similarity score for these two test tweets is 1.0. This training tweet is then considered as a retweet of the test tweet washington, even though it returns #wsuv instead of #DC.

As previously observed for HF-IHU in Fig. 11, kNN also shows that the no-retweet line plots are almost overlapped with the All-Dataset line plot, yet slightly less overlap than the HF-IHU. We further analyzed the difference simply by finding the percentage of matched hashtags due to the presence of retweets. We let $M_A$ denote the number of hashtag matches when the training set contains all data sets. We also let $M_{NR}$ denote the number of hashtag matches when the training set contains no retweets. Then, the percentage of hashtag *matches* due to the presence of retweets, $M_{R}$, is computed as follows:

15 $$\begin{aligned} M_{R} = \frac{M_A - M_{NR}}{M_A}. \end{aligned}$$

**Fig. 13:**
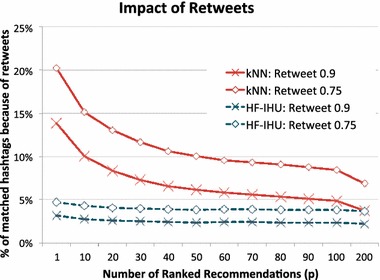
Percentage of matched hashtags due to the presence of retweets depending on the ranking method

Figure 13 shows the result with four line plots: HF-IHU with $r=0.9$, HF-IHU with $r=0.75$, kNN with $r=0.9$, and kNN with $r=0.75$, where $r=0.9$ means that all tweets scored greater than 0.9 in Cosine similarity are considered retweets. We can observe that kNN is affected by the presence of retweets in the training set, while our HF-IHU is much more resistant. At $x=1$, kNN with $r=0.9$ shows that approximately 14 % of hashtags were reproduced because of retweets, and kNN with $r=0.75$ slightly increases to 20 % because more tweets are considered retweets. Both kNN line plots consistently decrease as more hashtags are returned. At $x=200$, for example, the number of matched hashtags increases, but it is much less probable that those hashtags come from retweets. On the other hand, both HF-IHU line plots show that the percentage of matched hashtags due to retweets is consistently under 5 %, regardless the changing number of high-scored hashtags, thus not affected by the presence of retweets in the training set.

#### Case study: recommendations for users

As the last element of our analysis, we want to have some qualitative evidence of the effectiveness of the recommendation system. To this end, we retrieved a list of the most prolific users (tweeted most frequently) in our data set. From this list, we then selected three sample users with clear interests: *@XboxSupport*, *@jewishblogger*, and *@freeprojectinfo*. *@XboxSupport* is a twitter account set up to provide support for XBox users. *@jewishblogger*, according to their profile page, are a “worldwide leader in Jewish and Israeli blogs”. *@Freeprojectinfo* tweets about freelance job postings. These sample users were selected because their tweets seem to focus on a relatively narrow range of topics, and thus we should be able to manually validate recommendations provided by our system with a reasonable amount of confidence.

Given the tweets by each sample user as input, Table 3 lists the top 10 recommended hashtags ranked with our proposed method. For each recommended hashtag, we determined if the tag was clearly related to the topics covered based on the account profile, and if so we marked that hashtag as a *hit*. When a recommended hashtag had no intuitive semantic value, we performed a web search to provide a first-order approximation on the meaning associated with the tag before determining whether it qualified as a hit.

**Table 3:** Top 10 recommended hashtags ranked with HF-IHU

*@XboxSupport*		*@jewishblogger*		*@freeprojectinfo*	
#vuze	$\bullet$	#israel	$\bullet$	#jobs	$\bullet$
#kinect	$\bullet$	#jewish	$\bullet$	#freelance	$\bullet$
#egypt		#obama		#webdevelopment	$\bullet$
#jan25		#israeli	$\bullet$	#job	$\bullet$
#jobs		#telaviv	$\bullet$	#egypt	
#fb		#synagogue	$\bullet$	#design	$\bullet$
#sissyboys		#gasztro	$\bullet$	#jan25	
#xbox	$\bullet$	#parashat	$\bullet$	#fb	
#ff		#jan25		#seo	$\bullet$
#nowplaying		#jerusalem	$\bullet$	#wordpress	$\bullet$
Hits	3		8		7

Table 3 shows that the recommended hashtags ranked with HF-IHU include many pertinent hashtags for *@jewishblogger* and *@freeprojectinfo*, but only a few relevant hashtags for *@XboxSupport*. #vuze was the only tag that did not have an intuitive semantic value. A cursory search indicates that Vuze is a program that allows users to stream music and videos through devices, such as XBox consoles, so it was deemed a hit.

**Table 4:** Top 10 recommended hashtags ranked with kNN

*@XboxSupport*		*@jewishblogger*		*@freeprojectinfo*	
#codysimpsonu...		#codysimpsonu...		#codysimpsonu...	
#backintheday		#cruzazul		#cruzazul	
#nowplaying		#lastfm		#lastfm	
#fb		#nowplaying		#nowplaying	
#np		#news		#news	
#ff		#followmejp		#followmejp	
#mentionke		#zodiacfacts		#zodiacfacts	
#zodiacfacts		#magistream		#magistream	
#bkstage		#sougofollow		#sougofollow	
#codysimpson		#win		#win	
Hits	0	0		0	

Unlike the hashtags recommended by HF-IHU, kNN fails to identify *any* intuitively salient tags for our three sample users (Table 4). Moreover, most of recommended hashtags by kNN are in the top 50 popular hashtags. As observed in the evaluation with retweets, the performance of kNN method is directly affected by retweets in the data set. Since there are more terms that were tweeted with popular hashtags, it is more probable that tweets containing popular hashtags score high with Cosine similarity.

Because the purpose of hashtag recommendation is to introduce new hashtags to users, it is worth examining the novel hashtags (i.e., those not already used by the user). Table 5 shows the result of the HF-IHU method when we remove recommended hashtags that were found in any of the user’s prior tweets.

**Table 5:** Top 10 recommended #hashtags that are not used in user’s tweets

*@XboxSupport*		*@jewishblogger*		*@freeprojectinfo*	
#vuze	$\bullet$	#gasztro	$\bullet$	#jobs	$\bullet$
#kinect	$\bullet$	#parashat	$\bullet$	#freelance	$\bullet$
#egypt		#jerusalem	$\bullet$	#webdevelopment	$\bullet$
#jan25		#egypt	$\bullet$	#job	$\bullet$
#jobs		#holocaust	$\bullet$	#egypt	
#fb		#judaism	$\bullet$	#design	$\bullet$
#sissyboys		#mentionke		#jan25	
#xbox	$\bullet$	#jew	$\bullet$	#fb	
#ff		#talmud	$\bullet$	#seo	$\bullet$
#nowplaying		#nowplaying		#wordpress	$\bullet$
Hits	3		8		7

Both *@XboxSupport* and *@freeprojectinfo* did not use many hashtags in their tweets, resulting in no changes in the correlation rate. Interestingly, although the recommendations for *@jewishblogger* did change, their overall hit score stayed the same. This lends additional evidence to the quality of recommendations provided by our approach. Although popular hashtags sometimes include such important topics that every user should be aware of, #egypt and #jan25 during the Egypt revolution, for example, many of them still consist of frequently used twitter terms such as #ff(short for follow-friday), #nowplaying (tagged with songs), and others. Table 6 shows the recommendations when we exclude any of the top 30 most popular hashtags.

**Table 6:** Top 10 recommended #hashtags not including top 30 most popular #hashtags in the data set

*@XboxSupport*		*@jewishblogger*		*@freeprojectinfo*	
#vuze	$\bullet$	#gasztro	$\bullet$	#freelance	$\bullet$
#kinect	$\bullet$	#parashat	$\bullet$	#webdevelopment	$\bullet$
#sissyboys		#jerusalem	$\bullet$	#job	$\bullet$
#xbox	$\bullet$	#holocaust	$\bullet$	#design	$\bullet$
#xbox360	$\bullet$	#judaism	$\bullet$	#seo	$\bullet$
#taddei		#jew	$\bullet$	#wordpress	$\bullet$
#job		#talmud	$\bullet$	#lukewilliamss	
#5		#bethaderej	$\bullet$	#html	$\bullet$
#coupon		#sm		#css	$\bullet$
#deals		#orangotag		#marketing	$\bullet$
Hits	4		8		9

Without hashtags already used by the user and the top 30 popular hashtags in the recommended hashtags with our HF-IHU method, the hit rate increased or stayed the same for all three users; again adding evidence as to the quality of the recommendations. Table 7 shows, on the other hand, that removing popular hashtags from the recommended hashtags by the kNN method simply lists other popular hashtags from the high-scored hashtags list. Further note that, as before, the hit rate of kNN is 0 for all users.

**Table 7:** Top 10 recommended #hashtags ranked by the KNN method, not including top 30 most popular #hashtags in the data set

*@XboxSupport*		*@jewishblogger*		*@freeprojectinfo*	
#codysimpsonust$\dots$		#codysimpsonust$\dots$		#codysimpsonust$\dots$	
#backintheday		#cruzazul		#cruzazul	
#zodiacfacts		#lastfm		#lastfm	
#bkstage		#zodiacfacts		#zodiacfacts	
#codysimpson		#magistream		#magistream	
#thatswhatiwant		#sougofollow		#sougofollow	
#bears		#ebay		#win	
#goodwoman		#sagittarius		#ebay	
#packers		#codysimpson		#sagittarius	
#cruzazul		#qanow		#qanow	
Hits	0		0		0

### Performance evaluation

In "Index generation and ranking" section, we described our indexing structures and generation algorithm in Map-Reduce. To carry out these algorithms, we installed Hadoop version 2.6.2 on a MacOS X machine running on a 3.2 GHz quadcore Intel Xeon CPU, 8 GB RAM, and a 2 TB hard disk. Because the corpus data set (3 GB) is not prohibitively large, we can achieve good performance by simply run Hadoop in pseudo-distributed mode. The corpus data were split into 64 MB blocks (Hadoop default), and loaded into the Hadoop Distributed Filesystem (HDFS). The number of splits defines the number of map tasks that are spawned by the runtime. Specifically, the Hadoop mapreduce system will create $3\, \text{GB}/ 64\, \text{MB} = 48$ tasks. Each task is scheduled on a CPU core when it becomes available.

**Fig. 14:**
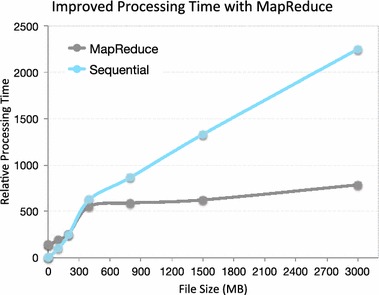
Map-Reduce vs. sequential implementation

We compared the Hadoop implementation against a sequential implementation that is executed on the same machine. Figure 14 shows the performance comparisons over increasing sizes of our corpus.

The speedup of Map-Reduce is not apparent for smaller data sets, which is expected due to the overhead costs (initialization and cleanup routines) of invoking Hadoop. However, these costs are amortized when the corpus size reaches around 1 GB and beyond. In the final experiment, we processed a 3-GB corpus and observe roughly a $3.2\times$ speedup over sequential. This result is expected, over a 4-node pseudo-distributed execution.

## Related work

As the number of micro-blog users increases, Twitter has become one of the most powerful medium generating millions of free-form tweets per day, and many researchers and industries have conducted extensive analysis of micro-blogs data since Twitter launched in 2006. Most of the research mainly focus on data organization and retrieving important information. However, there are four bodies of work that overlap significantly with our project. We discuss each below.

### Keyword extraction

Due to rapidly growing population of social media, majority of today’s businesses are participating in social media marketing and Twitter has been one of the most popular platforms for marketers [16].

As a potential commercial application, Wu et al. designed a system for automatically generating personalized annotation tags to label users’ interests based on users’ tweets [17]. In their pre-processing stage, they applied the Stanford POS tagger so that only nouns and adjectives are selected as valid keyword candidates, and compared TF-IDF ranking and TextRank [18]. Given a collection of tweets for a user, TF-IDF scores each term for its term frequency normalized for length and the IDF weighs down the term’s score if it appears in many users’ tweets. In the TextRank method, a collection of tweets by one user is modeled as a graph where each term is represented by a vertex. Both methods were experimented for approximately 11,000 Twitter users, and their tagging results were evaluated by three human evaluators judging based on the relevancy between the recommended tags and user’s interests. The experimental results show that TextRank slightly outperformed TF-IDF ranking, but both methods resulted in high precision (approximately 60 %). Michelson et al. also present a method to discover user interests by analyzing user tweets [19]. They leverage Wikipedia as a knowledge base to generate a sub-tree of candidate categories associated with the key entities in tweets, and those retrieved categories are then ranked based on the frequency and the category’s level in the sub-tree. Four sample users, 300 tweets per user in average, were selected for the experiments, and they evaluated whether the retrieved topics are relevant to the tweet’s actual topics that are manually discovered by reading through their tweets. Although the stated purpose of this study is to generate topic profiles for Twitter users, the discovered topics such as SPORT IN ENGLAND and CHICAGO CUBS can be used for marketing.

Although their work is similar to ours in a sense that we both try to find user interests, Wu et al. extract relevant terms within user’s own tweets and Michelson et al. retrieve candidate topics from Wikipedia, whereas we focus on finding topics that ideally are new to users and utilizing the real-time information retrieved from the Twitter network.

### User classification

Pennacchiotti et al. present a method to classify Twitter users in various classes such as political orientation, ethnicity and business fan detection (e.g. Starbucks fans) analyzing four general feature classes: user profile, tweeting behavior, tweet contents and user network [20]. Many studies proposed various mechanisms to detect spammers in social media [21–23]. As example, Magno et al. [21] provide the spammer attributes to differentiate spammers and non-spammers and their experiments applying a supervised machine learning method results in high classification accuracy and low misclassification of non-spammers. Romero et al. developed an algorithm to measure the influence and passivity of all the users in the Twitter network, and found that the majority of users with high passivity tend to be spammers and robot users [24].

These works focus on analyzing the features for the specific classification of users and apply those features to find user classification. Since our focus is on finding the most relevant hashtags for tweets, our ranking method does not implement conditions to find any specific features in users or tweets.

### Category recommendation

Sriram et al. present a method to classify tweets to a predefined set of classes such as *news*, *events*, *opinions*, *deals*, and *private messages* [4]. Their approach relies on features derived from the tweet contents. A news feature, for example, may be absence of emoticons and slang words, and presence of a currency symbol may be a feature for deal. Similarly, Esparza et al. [5] suggest hashtags in five pre-defined categories (*movies*, *books*, *music*, *apps* and *games*) focusing more on the textual contents of tweets to encourage the use of hashtags. They manually created a category-term map, and rank each category with TF-IDF where TF denotes the term occurrence in a list of terms for a category and IDF denotes the frequency of occurrence of the term in all categories.

The purpose of category recommendation systems is to suggest topics for user tweets so that users can group their tweets into specific topics to facilitate easier search. In contrast, one of the motivation in our work is to help users discover new topics by suggesting personalized hashtags; therefore, our approach does not limit the number of candidate hashtags by specific topics.

### Hashtag recommendation

Most related to our work is the class of hashtag recommendation systems. Zangerle et al. compare three different hashtag ranking methods in Recommending #-Tags in Twitter [7]. Receiving a user’s tweet, they first find similar tweets in their data set using TF-IDF and retrieve a set of candidate hashtags that appeared in these most similar tweets. They rank the hashtags based on the overall popularity of candidate hashtags, the frequency of candidate hashtags within the most similar tweets, and the similarity score of the most similar tweets. The reported results show that the third method performed the best in recommending hashtags. Their approach solely relies on tweets’ similarities and those hashtags occurred in the most similar tweets are recommended to users, whereas our approach more focuses on terms in tweets and the relevance of those terms to candidate hashtags.

Kywe et al. proposed a method that recommends hashtags retrieved from similar users and/or similar tweets [15]. They compute the preference weight of a user towards a hashtag in the data set using the TF-IDF scheme, and then select the top *n* users who scored high in cosine similarity between a user and another user. The top *m* similar tweets are selected in a similar manner. Their approach basically adds more hashtags (used by similar users) to the list of candidate hashtags retrieved by the method proposed by Zangerle et al. However, when target users have never used hashtags before, the recommendations only include hashtags from similar tweets. Although user similarity is taken into account in this method, many of recommended hashtags may be from similar tweets because majority of tweets do not contain hashtags [15, 25], and also their approach still focuses on similarities in terms and used hashtags, while our approach does not rely on similarities.

Godin et al. point out the challenge of ranking hashtags based on the tweet’s similarity and recommending hashtags existing in similar tweets due to the sparseness of hashtags [6]. To combat this challenge, their approach focuses on detecting hidden topics for the tweets and then suggests the use of those general topics as hashtags using a latent dirichlet allocation (LDA) model to facilitate better search. Although both our approach and their approach take into account the data sparseness of micro-blog data, the fundamental difference is that their approach limits the suggestions to general topics. Our approach rather attempts to retrieve relevant and emerging hashtags in the data set.

Dovgopol et al. propose a recommendation model based on *k*-Nearest Neighbor and Naive Bayes [26]. They first find three most important words in a target tweet using inverse document frequency (IDF) and use all tweets that contain at least one of those three words to speed up the system. Bayes’ Theorem scores hashtags by the probability of co-occurring with each term in a tweet, and *k*-Nearest Neighbor score is the number of hashtags that occurred in similar tweets. Their comparison result showed that the hybrid model performed slightly better than using only one model. Our method, however, delves into the impact of retweets as they can be identical to a target tweet and the hashtags in retweets often receive the highest score. In our method, we removed retweets from the training set so that the recommendation results do not get affected by retweets.

Lu et al. propose a model for hashtag recommendation which collects time-sensitive latent topics from tweets by combining the Topics-over-Time (TOT) Model with the mixed membership model (MMM) [27]. They estimate the topic mix of given tweets based on words and time stamp, that determine the distribution of words in the tweet, and recommend words with high probabilities of occurring in the target tweet as hashtags. Their result show that the difference between TOT-MMM and similarity-based approach with a time-clustering effect (SIM-T) is not significant; however, TOT-MMM combined with SIM-T yielded the best performance among all approaches considered in their study. The main focus of their model is to capture timely topics rather than performance improvements in terms of computational speed, while one of our goals is the ability to quickly work with a large Twitter data set.

## Conclusion and future work

The objective of this paper was to implement an effective hashtag recommendation system that automatically suggests a list of personalized hashtags emerging real-time for Twitter users.

Inspired by classic information retrieval approaches, we proposed the use of an inverted-index data structure to store two frequency maps that are be built prior to performing the hashtag ranking. By leveraging these inverted-indices, the term/hashtag look-ups are performed in *O*(1) time, and thus we achieve faster and more effective search of associated hashtags for a term and vice-versa. We showed a Map-Reduce-based algorithm to scalably build these inverted-indices over large Twitter data sets. We proposed a ranking method, *Hashtag Frequency-Inverse Hashtag Ubiquity* (HF-IHU), which is a variation of the TF-IDF weighting scheme to score hashtag relevancy while also taking into account data sparseness of Twitter data set.

Our experiments on a large Twitter data set demonstrated that our proposed method performed better than other methods that rely only on hashtag popularity and tweet similarity. Further experiments clearly showed that our performance is more stable and reliable than ranking based on tweets’ content similarity. Finally, we conducted experiments on the top 10 high-scored hashtags. Compared with a ranking method based on cosine similarity, the experiments exhibited that our system consistently assigned high score on hashtags that interests the user.

While our research has demonstrated promising results on recommending personalized hashtags, the scope of the research can be extended in several other directions in the future. We discuss the most prominent. There exist several studies on sentiment analysis for the domain of microblogs. Text sentiment could potentially be used to detect user’s interests more accurately and make better hashtag recommendations. Some previous efforts show sentiment analysis on the whole tweet [28, 29]. Zhang et al. propose sentiment analysis at the entity level [30]. We could exploit this analysis so that entities with positive sentiment have a greater impact on the hashtag recommendations than entities with negative or no sentiment. For example, provided “*I bought iPad yesterday and love it* :-)”, hashtags relevant to iPad score high because the entity iPad is positive.

Liu et al. presented a news recommendation system that leverages collaborative filtering and was improved by adding information filtering [31]. These synergistic methods could also be used for hashtag recommendation as in our current work. Tweet content analysis in our method could be viewed as information filtering, and we could add collaborative filtering based on a users’ subscription (i.e., the users they follow). Research on Twitter users’ motivation found that there are many users who are categorized as “information seekers: those who post rarely, but follow other users regularly.” [32]. Even though collaborative filtering increases computational complexity, it may dramatically improve recommendations when an individual user has relatively few posts as is the case with information seekers.

Finally, we note that hashtag recommendation may be relevant for more use-cases than our work has so far explored. In particular, a recommendation system could be used to recommend hashtags for a particular tweet from within a user’s own lexicon. This could be done, for example, by limiting recommendations to the set of hashtags that a user has previously applied to her tweets.

## Footnotes

1 Twitter-corpus-tools, http://www.twittertools.cc.
